# Constructing gene regulatory networks for long term photosynthetic light acclimation in *Arabidopsis thaliana*

**DOI:** 10.1186/1471-2105-12-335

**Published:** 2011-08-11

**Authors:** Cheng-Wei Yao, Ban-Dar Hsu, Bor-Sen Chen

**Affiliations:** 1 Lab of Control and Systems Biology, Department of Electrical Engineering, National Tsing Hua University, Hsin-Chu, 300, Taiwan; 2 Department of Life Science, National Tsing Hua University, Hsin-Chu, 300, Taiwan

## Abstract

**Background:**

Photosynthetic light acclimation is an important process that allows plants to optimize the efficiency of photosynthesis, which is the core technology for green energy. However, currently little is known about the molecular mechanisms behind the regulation of the photosynthetic light acclimation response. In this study, a systematic method is proposed to investigate this mechanism by constructing gene regulatory networks from microarray data of *Arabidopsis thaliana*.

**Methods:**

The potential TF-gene regulatory pairs of photosynthetic light acclimation have been obtained by data mining of literature and databases. Following the identification of these potential TF-gene pairs, they have been refined using Pearson's correlation, allowing the construction of a rough gene regulatory network. This rough gene regulatory network is then pruned using time series microarray data of *Arabidopsis thaliana *via the maximum likelihood system identification method and Akaike's system order detection method to approach the real gene regulatory network of photosynthetic light acclimation.

**Results:**

By comparing the gene regulatory networks under the PSI-to-PSII light shift and the PSII-to-PSI light shift, it is possible to identify important transcription factors for the different light shift conditions. Furthermore, the robustness of the gene network, in particular the hubs and weak linkage points, are also discussed under the different light conditions to gain further insight into the mechanisms of photosynthesis.

**Conclusions:**

This study investigates the molecular mechanisms of photosynthetic light acclimation for *Arabidopsis thaliana *from the physiological level. This has been achieved through the construction of gene regulatory networks from the limited data sources and literature via an efficient computation method. If more experimental data for whole-genome ChIP-chip data and microarray data with multiple sampling points becomes available in the future, the proposed method will be improved on by constructing the whole-genome gene regulatory network. These advances will greatly improve our understanding of the mechanisms of the photosynthetic system.

## Background

Life on earth is dependent on energy derived from the sun, and photosynthesis is the only biological process able to harvest this energy. Plants must maintain high photosynthetic efficiency to ensure sufficient energy for survival and seed production for the next generation. As plants are sessile organisms and cannot escape environmental changes that directly affect photosynthetic light reactions, they have evolved regulatory mechanisms that optimize photosynthetic electron transport to acclimate the photosynthetic process to the prevailing environment [1,2]. Changes in the intensity and spectral quality of light received by plants beneath a tree canopy or within dense plant population contribute to imbalance in the excitation of energy distribution between photosystem II (PSII) and photosystem I (PSI). Because PSI and PSII work electrochemically in series, if either of the two photosystems is imbalanced the redox state of the electron transport chain components is changed, decreasing the efficiency of electron flow [3]. Such imbalances are counterbalanced by two different acclimation responses: state transition, which is a short term response, and adjustment of photosystem stoichiometry, which is a long term response [1]. The focus of this study is on the regulatory mechanisms of the long term response (LTR) to fluctuating light quality in the nucleus.

**Figure 1:**
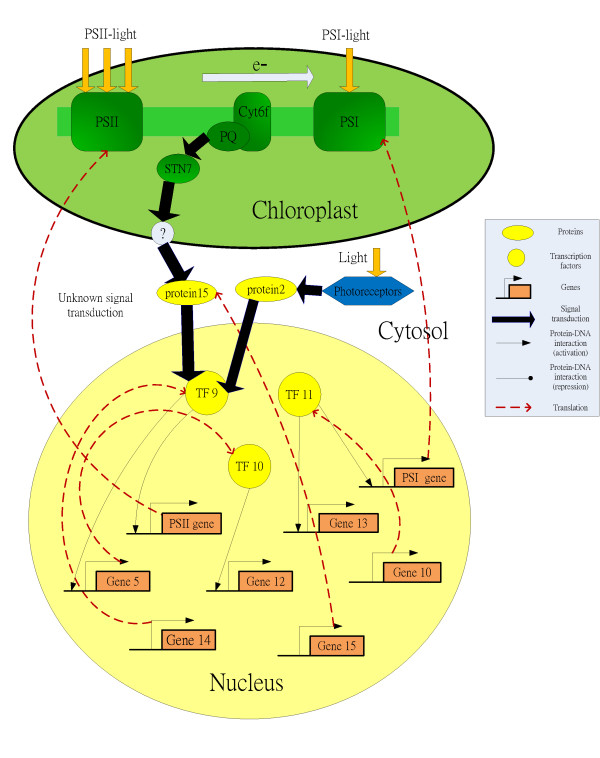
**Effect of light quality on gene expression**. The three cell compartments are depicted schematically: chloroplast (green), cytosol (white) and nucleus (yellow). The integrated cellular network consists of two subnetworks. One network is the signaling transduction pathways in the cytosol and the other network is the gene regulatory network for transcriptional regulation in nucleus. An important mediator for PQ pool redox states is the thylakoid kinase STN7. STN7 kinase transmits the decisive signal to the nucleus, resulting in the ensuing regulation of the relative amount of each of the photosystems. However, the mechanism by which the redox signal is transmitted from the chloroplast double membrane into the cytosol is poorly understood. In this study, the gene regulatory network (the protein-DNA interaction) in the nucleus is constructed under different retrograde signals originating from different redox states in the PQ pool. The light environment is perceived by cytosolic photoreceptors. Although both PQ pool and photoreceptor systems report changes of ambient light environment by different signal transduction pathways to the nucleus, some common TFs in nucleus may be employed simultaneously in different light-related systems to respond to the prevailing environment.

Adjustment of photosystem stoichiometry is a long term response that occurs on a time scale of hours to days. It re-distributes excitation energy between PSII and PSI mainly by reconfiguring the relative amount of the two photosystems, enabling them to optimize light utilization [2-6]. Imbalances in components of the photosynthetic electron transport chain are sensed within the chloroplast. The change in redox state of the plastoquinone (PQ) pool of the electron transport chain is the critical regulatory signal source for transcriptional control. This change delivers a signal to the nucleus and chloroplast to modulate the expression of photosynthetic genes encoding the PSII and PSI proteins (Figure 1). Such redox signals from chloroplast to nucleus are the so-called retrograde signals, in which photosynthetic efficiency is thought to be a sensor for fluctuations in the environment. The retrograde signals represent a functional feedback control which directs the expression of genes in the nucleus to respond to disturbances from the surrounding environment [4]. This long term response process has been relatively well characterized at the biophysical level, however the acclimation process remains poorly understood at the bio-molecular level. For instance, most subunits of photosystems are encoded in the nucleus and require special regulation, but these regulatory processes are not yet understood. The primary aim of this study is to use a systems biology approach to investigate the molecular mechanisms of photosynthetic light acclimation by constructing nuclear transcriptional gene regulatory networks under different PQ pool redox states. This has been achieved using time series microarray data of *Arabidopsis thaliana *under an artificial light system. The artificial light system mimics light conditions by preferentially exciting PSI or PSII to induce more reduced or oxidized states of the PQ pool by shifting from PSI light to PSII light, or from PSII light to PSI light.

The foremost purpose of the study of molecular biology is to decipher the mechanisms behind the biological processes of cells. Recently, the development of high-throughput genomic tools such as DNA microarray [7,8] and chromatin immunoprecipitation-DNA chip (ChIP-chip) data [9,10] have provided comprehensive information about gene expression activity and potential interactions between transcription factors and genes. Although this data catalogs significant patterns of the dynamic expression of thousands of genes, identifying the characteristics of a biological process under a specific condition among vast amounts of experimental data is still an extremely difficult task. These vast amounts of data emphasize the need for systematic approaches that enable the computational reconstruction of dynamic transcriptional regulatory networks [11,12]. Systematic approaches are based on integrating computational methods with high-throughput and genome-wide data to inspect the nodes and edges of gene networks [13]. These descriptive and mathematical models for gene regulatory networks can uncover significant dynamic properties of biological systems from their responses to internal and external signals. Consequently, the use of a systematic approach to reverse engineering has become an extremely important tool for identifying gene regulatory networks via gene expression data.

Various approaches of reverse engineering have been used to identify plausible gene networks, including clustering algorithms, ordinary differential equation (ODE) models and Bayesian networks (BNs), which have been extended to construct dynamic Bayesian networks (DBNs) [14,15]. Clustering algorithms have been used to find co-regulated genes based on gene co-expression properties. They are therefore suitable to find a set of genes that potentially have the same regulators or functional modules. Besides, both DBNs and ODE network models have previously been used successfully to model dynamic processes with time series microarray data. Furthermore, some studies [16] have incorporated many other types of heterogeneous data to reconstruct gene networks. However, in the plant model, *Arabidopsis thaliana*, the types of heterogeneous data are limited, making it difficult to apply a systematic approach to construct gene networks. In a previous study [17], a novel Bayesian network-based algorithm was adapted to construct a gene regulatory network for *Arabidopsis thaliana *from a large number of separate microarray experiments without heterogeneous data. JIAO QingJu, et al. [18] constructed a gene regulatory network for *Arabidopsis thaliana *by utilizing promoter analysis to predict plausible transcriptional relationship. They were able to identify large-scale gene regulatory networks which included photosynthetic genes but did not mention the central molecular mechanism of photosynthesis. In this study, we have analyzed the molecular mechanisms of photosynthetic light acclimation by adapting a systems biology approach to construct gene regulatory networks using prediction database and time series microarray data. We constructed two gene regulatory networks under different PS-light shifts to gain insight into the mechanism of photosynthetic light acclimation. Such gene networks based on our algorithm can find the TFs integral to regulating the photosynthetic light acclimation response.

Here we propose a systematic approach based on the dynamic gene regulatory model and time series microarray data to gradually refine the rough gene regulatory network. The first requirement is to identify the candidate TF-gene interactions and construct a rough gene regulatory network. Due to the limit of ChIP-chip data and network-related studies on *Arabidopsis thaliana*, we have used Plant Promoter Analysis Navigator (PlantPAN) [19] to compensate for this insufficiency by inferring potential TF-gene interactions from data mining of the literature and databases. Pearson's correlation between each candidate TF-gene pair is then computed to gain a rough gene regulatory network. This rough gene regulatory network is added to using an efficient dynamic model of gene regulation [20,21] and time series microarray data [22], thus producing a dynamic gene regulation system. As a final step, maximum likelihood system identification methods [23] and Akaike information criterion (AIC), a method to detect the order of the dynamic gene regulatory system [23], are employed to identify kinetic parameters in the dynamic gene regulation model and to delete regulatory genes that do not have a significant influence on the target gene in the rough gene regulatory network. This method of applying an algorithm which combines system identification and AIC to reduce the insignificant interactions of rough gene regulatory and protein-protein interaction networks has been used successfully in many studies, including the study of a putative gene regulatory network of systemic inflammation in humans [21], a biofilm-related gene regulatory network in *Candida albicans *[24], *a *cancer-perturbed protein-protein interaction [25] and a protein-protein interaction under several stresses in *Saccharomyces cerevisiae *[26]. These results were validated by evidence within literature, thus demonstrating that such systems biology approaches provide a powerful and flexible tool which can be used for different species under different conditions.

The time series microarray data is the profile of each gene expression at several specific time points under a specific stress or condition. Using the dynamic model of gene regulation and system identification method alongside time series microarray data, false positive interactions between transcription factors and target genes can be removed to obtain a refined gene regulatory network. This refined gene regulatory network depicts a real gene regulatory network under photosynthetic light acclimation responses, as confirmed by real microarray data. Therefore, pruning the rough gene network based on a systematic approach and time series microarray data of *Arabidopsis thaliana *can produce a refined gene regulatory network which deciphers photosynthetic light acclimation at both bio-molecular level and a system-wide level.

## Method

In this study, the construction of gene regulatory networks for long-term photosynthetic light acclimation in *Arabidopsis thaliana *can be divided into two stages. During the first stage, the candidate TF-gene interactions are identified by data mining of the literature and PlantPAN [19] to develop a rough gene regulatory network. During the second stage, the rough gene regulatory network is pruned by the maximum likelihood system identification and AIC system order detection. These pruning methods have been supplemented with time series microarray data to obtain a refined gene regulatory network.

### Data used for analysis

We have used previous microarray data [22] as our mRNA expression profile. Plants acclimated to PSI or PSII lights were shifted to the alternate condition to induce either a strong reduction (PSI-to-PSII light shift) signal or a strong oxidation (PSII-to-I light shift) signal. The LTR to such shifts is followed by collecting samples prior to the shift (t = 0 control) and at 0.5, 2, 8, and 48 h thereafter. Transcript profiles from those samples were obtained using an established DNA filter array with gene-specific tags (GSTs) for nuclear genes encoding chloroplast proteins. The filter array, which carries 3292 GSTs, including 2661 nuclear genes for chloroplast proteins and 631 genes encoding for non-chloroplast proteins, was used to test the impact of light-induced redox signals on their expression.

PSI-to-PSII and PSII-to-PSI experiments were conducted separately in neighboring growth cabinets of the same climate chamber. Plants were acclimated to PSI and PSII light. Following this acclimation period the plants were moved to the alternate light. Three independent samples were harvested at each indicated time point for RNA preparation. Total RNA was isolated from 200 to 500 individual plants per sample. Three independent hybridization experiments were performed for each time point, with different filters and independent radioactively label cDNA probes, thus minimizing variation between individual plants, filters, and probes.

### Stage I: Construction of a rough gene regulatory network for photosynthetic light acclimation

At this stage, the construction of a rough gene regulatory network is performed in the following four steps:

#### Step 1

The target gene pool that is potentially related to the long-term response of photosynthetic light acclimation is determined by data mining of the literature and database. According to previous studies [2], the long-term response of photosynthetic acclimation can counteract an imbalance by adjusting the relative amount of the two photosystems. Therefore we explore the Kyoto Encyclopedia of Genes and Genomes (KEGG) database to retrieve the nuclear genes encoding photosystems or light-harvesting protein complexes. From this database, we also select the genes involved in the photosynthetic light reaction pathway. Moreover, the tentative target genes of the long term photosynthetic light acclimation identified in [27] by analyzing photosynthetic impairment in varieties with double mutated gene are added into the target gene pool. Our final aim is to select candidate regulators of the target gene pool to obtain the potential TF-gene interactions involved in photosynthetic light acclimation response.

#### Step 2

Each target gene is imported to PlantPAN, a plant promoter analysis navigator which recognizes candidate binding sites of transcription factors in the promoter sequence of target gene, from -1000 (upstream) to +100 (downstream). At this stage, the candidate TFs predicted by the PlantPAN database could be considered candidate regulators for the target genes. Any candidate TFs that are not present in the original target gene pool should be added to it in order to find their regulators iteratively. The iterative procedure is discontinued when no new interactions are generated from the PlantPAN database. Based on predictions of the PlantPAN database, it is possible to obtain the potential TF-gene interaction pairs involved in photosynthetic light acclimation response. However, the potential binding of a TF does not imply that the TF regulates the gene in photosynthetic light acclimation. In order to reduce the false positive errors, it is necessary to refine the TF-gene binding pairs. This is achieved by using time series microarray data of photosynthetic light acclimation to confirm the existence of these transcriptional bindings.

At this point, we have obtained an extended target gene pool coupled with its corresponding TF-gene pairs. However, due to the limited microarray data used in this study, genes and potential regulators that aren't present in the microarray data probe must be removed. Following this removal, a new target gene pool is obtained, with 65 genes containing 7 TFs (see additional file 1) and the potential TF-gene pairs of each gene (see additional file 2).

#### Step 3

In a previous study [28], a TF and its target gene were said to have a positive or negative temporal relationship if the target gene's expression profile is similarly correlated with the TF's expression profile. The Pearson's correlation was computed between each TF-gene pair based on this assumption. The candidate TF-gene pairs were then ranked according to the absolute value of correlation. Finally, any TF-gene pair below 10% rank was deleted. Because the correlation value is only the first discrimination parameter in the overall procedure of gradual refinement, it is necessary to avoid missing any possible candidate TF-gene pair at this early stage. The primary aim in this step is to delete only the highly unlikely regulatory interactions.

#### Step 4

At this stage, the first selection of possible regulatory interactions is selected from the TF-gene pairs identified in Step 3. They are regarded as candidate TF-gene pairs with the potential to become transcriptional interactions in gene regulatory network under photosynthetic light acclimation. These candidate TF-gene pairs constitute a rough regulatory network. Refinement of this preliminary result requires more rigorous pruning methods.

### Stage II: Pruning the rough gene regulatory network through the use of a dynamic gene regulation model with system identification methods

#### Step 1

Dynamic gene regulatory model: the rough gene regulatory network constructed in the previous stage using data mining and the Pearson's statistical inferences is expected to contain some false positive TF-gene pairs. Therefore the rough gene network should be confirmed by gene expressions of microarray data. Gene regulations that cannot be matched by time profiles microarray data are deleted.

The dynamic gene regulation model is used to depict the transcriptional regulatory mechanism as a system with several regulatory genes as transcriptionary inputs and a target gene's expression as an output. This allows the dynamic transcriptional regulation of a target gene to mimic a subsystem in the rough gene regulatory network. The multi-input single-output stochastic dynamic gene regulation model is proposed as follows(1)

where *y *(*t*) represents the target gene expression level at time t and S represents the set of potential regulators of the target gene in the rough gene network and could be set as *S *= [*L*] = {1, 2,..., *L*}. For each potential regulator *i *∈ *S *, *b_i _*explains how the expression of target gene is affected by the regulatory ability of TF *i*. *β *is attributable to the degradation effect of the target gene itself and *k *denotes the basal molecular level. In addition, *ε*(*t*) represents a stochastic noise due to uncertainty within the model and measurement error of microarray data in the target gene. Here *ε*(*t*) is assumed to be a Gaussian noise with zero mean and unknown standard deviation. Because biological systems seem to exhibit nonlinear characteristics, we assume a sigmoidal regulatory binding relationship between the regulator and the target gene. Furthermore, *x_i _*(*t*) is the transcriptional regulatory function of TFs on their motif binding sites, as described by following the sigmoid function of mRNA expression profiles on their corresponding regulatory genes [29], where the sigmoid function denotes the threshold of binding for a TF on its promoter binding site for the transcriptional regulation in equation (1). *x_i _*(*t*) can be expressed as follows:(2)

where *r *denotes the transition rate of sigmoid function and *m_i _*denotes the mean of the TF gene expression profile.

#### Step 2

**Figure 2:**
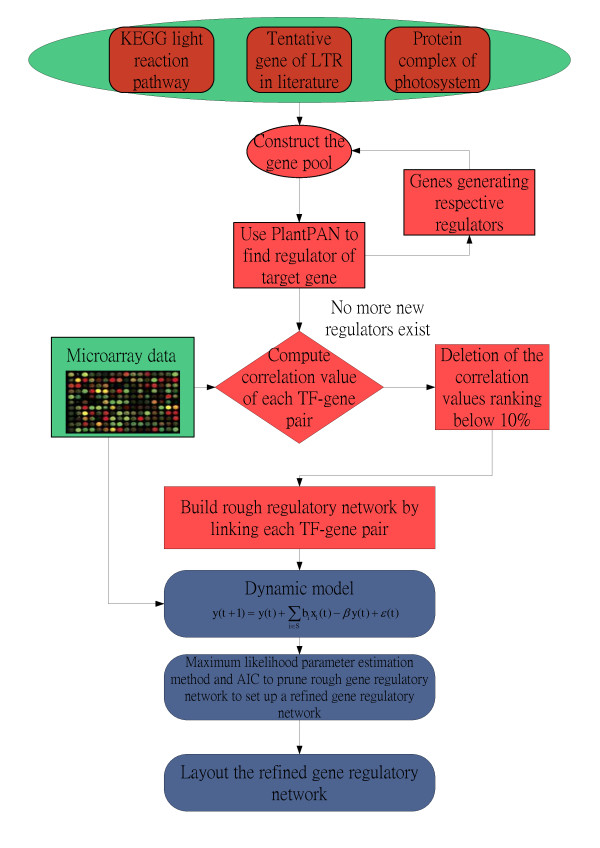
**The flow chart for constructing the gene regulatory network of photosynthetic light acclimation response**. The figure depicts the procedure for construction of the gene regulatory networks. Green is representative of data that is needed while red represents the procedure to construct the rough gene regulatory network for photosynthetic light acclimation (Stage 1). Blue is representative of the procedures which prune the rough gene regulatory network using the dynamic model with system identification method (Stage 2).

Refinement of the rough gene regulatory network: The model describes how upstream transcriptional factors modulate their target genes' mRNA expression through a transcriptional gene regulatory network. With the help of dynamic gene regulation model, the problem of network construction, which is difficult to decipher in biology, is transferred into a pre-identified system in engineering. The rough regulatory network is a large system which contains several subsystems. Each subsystem consists of a target gene and its regulatory TFs. The state of each target gene at different time points depends on the time series microarray data. This produces a large network system in which each state of the subsystem is known and the parameters of the subsystem have been estimated. These difficult parameter estimations are conducted using system identification methods, working with one target gene at a time. This estimation method involves combining the maximum likelihood (ML) parameter estimation method with the most parsimonious model order detection, Akaike Information Criterion (AIC), to identify the unknown element of the network system. In short, the parameters of system can be estimated using the maximum likelihood method and AIC can then delete any interactions without significant influence on this network system. These deletions are based on the results of the ML method and the degree detection of interactions in the network system. By approaching the network one target gene at a time, it is possible to identify all of the parameters of the rough gene regulatory network. Finally, to produce the pruned gene regulatory network, the overall gene regulatory system is deciphered dynamically and insignificant regulations are eliminated. This pruned gene regulatory network is close to real gene regulatory network in the cell. The overall flowchart of the proposed method is shown in Figure 2. Details of system identification methods are in the supplementary methods (see additional file 3).

In summary, the potential regulators of a target gene are first selected by the data mining of the literature and the PlantPAN database. Following this any highly unlikely regulatory relationship is removed via microarray with the help of Pearson's correlation. These possible relationships are finally pruned by time series microarray data of *Arabidopsis thaliana *via maximum likelihood system identification and Akaike's system order detection through a dynamic gene regulatory model. We have combined several algorithms and tools to improve the performance of the gene network construction of the photosynthetic light acclimation. Details of the proposed gene regulatory networks construction algorithm are shown in the Supplementary Methods (see additional file 3).

## Results

**Figure 3:**
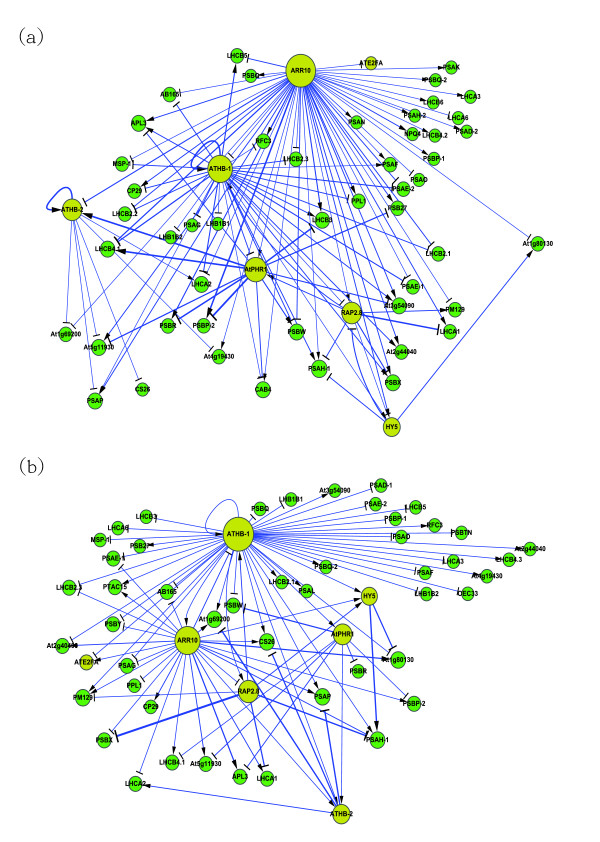
**The gene regulatory networks of long-term light acclimation response. **(a) Under reduced state of the PQ pool (PSI-to-PSII light shift). (b) Under oxidized state of the PQ pool (PSII-to-PSI light shift). The gene regulatory networks of long term light acclimation response visualized in a hierarchical form. Yellow represents the transcription factor and green represents the gene. The node size is proportional to the degree of each node and the edge width represents the magnitude of the regulatory ability *b_i _*in equation (1). This figure has been created by Cytoscape [30].

Based on data collected in Stage 1, we can construct a rough gene regulatory network of the photosynthetic light acclimation system. We then established a dynamic model for the rough gene network. By combining the system identification scheme and the parsimonious AIC method, the rough gene network was pruned using time series microarray data. According to the flow chart of Figure 2, it is possible to construct two refined gene regulatory networks, representing the PSI-to-PSII (reduced state of plastoquinone) and the PSII-to-PSI (oxidized state of plastoquinone) light shift conditions. These two gene regulatory networks have been rearranged and depicted by Cytoscape [30](Figures 3a and 3b). Figure 3a depicts the gene regulatory network under the reduced state of the PQ pool and identifies 55 nodes with 109 edges for the network. Alternatively, Figure 3b depicts the gene regulatory network for the oxidized state of the PQ pool and identifies 55 nodes with 92 edges for the network. The numbers of nodes, edges and highly connected hubs at different light conditions are shown in Table 1.

**Table 1:** Highly connected genes and their gene connectivities in two different light shift conditions

		PSI-to-PSII light shift	PSII-to-PSI light shift
Node		55	55

Edge		109	92

Gene name	AGI ID	Reduced state of the PQ	Oxidized state of the PQ

ATHB-1	At3g01470	33	48

ARR10	At4g31920	51	28

AtPHR1	At4g28610	14	8

RAP2.8	At1g68840	10	9

ATHB-2	At4g16780	10	7

HY5	At5g11260	6	6

Once the refined gene networks under different light shift systems have been constructed, the topology of the network can be examined. The degree of connection of each node in the two different conditions has been summarized [see additional file 4]. The results demonstrate that small-degree nodes are most abundant and high-degree nodes are relatively rare. This verifies that the networks constructed via our algorithm are scale-free, as opposed to random. Scale-free networks have smaller path-lengths than random networks and can tolerate random removal of nodes; however loss of the hubs may cause the network to collapse into clusters. Hence, smaller path-lengths ensure efficient reaction and higher tolerance of random mutation against internal and external perturbations [31].

There are two hubs, ATHB-1 and Arabidopsis Response Regulator 10 (ARR10), which can be regarded as highly connected hubs of signal transduction in the two different light conditions. Inactivation of these highly connected hubs by mutation may lead to collapse of the photosynthetic LTR system. Interestingly, the two hubs appear to have high importance for different light shift conditions. For the reduced state of the PQ pool, the gene ATHB-1 is the top rank and the gene ARR10 is the second rank in Table 1. However, for the oxidized state of the PQ pool, the ranking of these two genes is reversed. Within the two gene regulatory systems, some interactive genes can easily be removed whilst others can be easily added under different external stimuli. This concept is known as "weak linkage" in network theory [31]. "Weak linkage" structures enable the addition or removal of new or old processes to the existing core process using common versatile mechanisms that operate on diverse inputs and outputs [32]. As a consequence, "weak linkage" can improve the information exchange, signal transduction and network robustness in response to external stimuli [32]. Moreover, the most important interactions and information exchanges sometimes occur via nodes from otherwise unrelated networks implying that the non-hubs may play a critical role in gene regulation [31,32]. These non-hubs appear to have evolved by natural selection to improve the robustness of biological networks.

**Figure 4:**
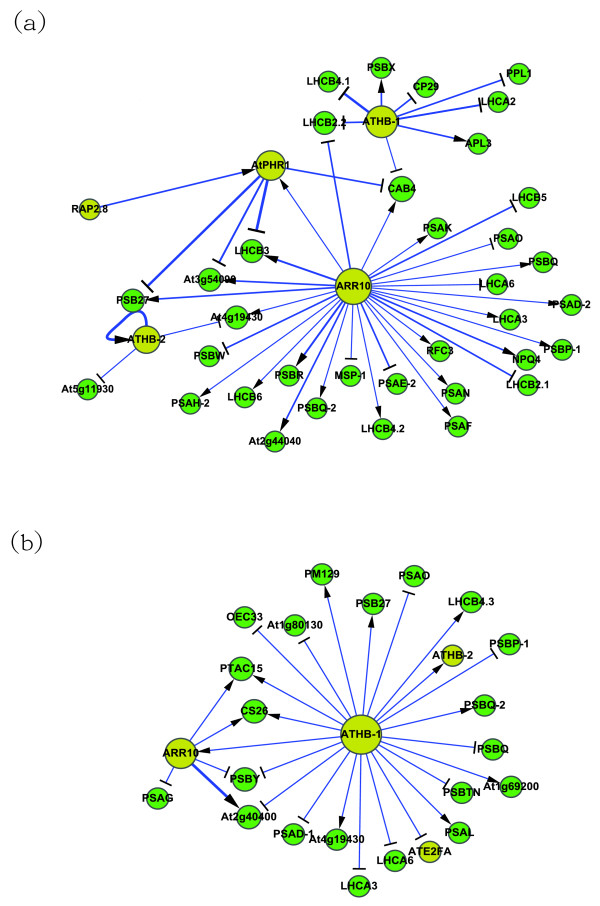
**The differential gene regulatory networks. **(a) Under reduced state of the PQ pool (PSI-to-PSII light shift). (b) Under oxidized state of the PQ pool (PSII-to-PSI light shift). Yellow represents the transcription factor and green represents the gene. The node size is proportional to the degree of each node and the edge width represents the magnitude of the regulatory ability *b_i _*in equation (1). This figure has been created by Cytoscape [30].

The gene regulatory networks in Figures 3a and 3b are too complex to distinguish which transcriptional interactions are present only in reduced state of the PQ pool or oxidized state of the PQ pool. Due to this, the differential gene regulatory networks (Figures 4a and 4b) have been obtained by comparing the network under PSI-to-PSII light shift conditions with the network under PSII-to-PSI light shift conditions. By matching the interactions found in both light shift conditions, we can identify the specific connections present only in PSI-to-PSII or PSII-to-PSI light shift conditions. Common interactions found in both conditions are considered to be a common subnetwork in both light conditions, which could be regarded as an inherent regulation under normal light conditions. The differential gene regulatory networks produced on elimination of this common subnetwork provide a clearer representation of which regulatory interactions effect photosynthetic light acclimation under the different redox states of the PQ pool. In Figure 4a, the differential regulatory network is found only in reduced state of the PQ pool, and it is clear that ARR10 is the main hub with the highest connectivity. In Figure 4b, the differential regulatory network is found only in oxidized state of the PQ pool, and ATHB-1 is the main regulator in this differential network. These observation of Figures 4a and 4b further confirm the importance of ATHB-1 and ARR10 in the opposite conditions. Principally, these observations indicate that ARR10 is responsible for the signaling transduction and transcriptional regulation of acclimation response under the reduced state of the PQ pool and that ATHB-1 is prominent under the oxidized sate of the PQ pool. Rather than investigating the entire gene regulatory networks, we can easily obtain the important information behind it in two antagonistic conditions by examining the differential networks.

**Figure 5:**
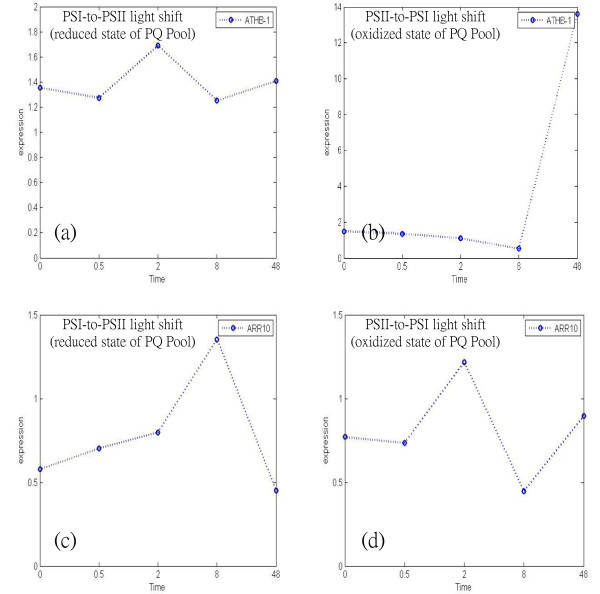
**The gene expression profile of ATHB-1 and ARR10**. (a) and (b) are the gene expression profiles of ATHB-1 after PSI-to-PSII (reduced state of PQ pool) and PSII-to-PSI (oxidized state of PQ pool) light shifts respectively; (c) and (d) are the gene expression profiles of ARR10 after PSI-to-PSII (reduced state of PQ pool) and PSII-to-PSI (oxidized state of PQ pool) light shifts respectively.

According to these results, the hubs ATHB-1 and ARR10 are identified with different significant status in opposite redox states of the PQ pool. As shown in [22], the initial transcriptional response occurred in the nucleus 30 min after PSI-to-PSII light shift. Alternatively, following the PSII-to-PSI light shift experiment, the initial transcriptional response occurred in nucleus 2 h after the shift. Figure 5 shows the time series expression profiles of both ATHB-1 and ARR10 in two different light shift conditions. Figures 5a and 5b demonstrate that the gene expression of ATHB-1 has an obvious change 8 h after the PSII-to-PSI light shift, but it does not appear to change following the PSI-to-PSII light shift, verifying that ATHB-1 is involved in the regulatory mechanism in the oxidized state of the PQ pool. The transcriptional expression of ARR10 exhibits a more noticeable change following the PSI-to-PSII light shift than following the PSII-to-PSI light shift in Figures 5c and 5d. Figure 5c shows ARR10 has significant change 2 h after the PSI-to-PSII light shift, verifying that ARR10 is involved in the regulatory mechanism under the reduced state of the PQ pool. Furthermore, transcription factor ARR10 is involved in the regulation of the chlorophyll biosynthetic process [33]. There are different types of chlorophyll in a chloroplast, chlorophyll a and chlorophyll b, both of which absorb different light spectra. Due to the treatment of PS-light shift, certain spectrum light is deficient which is not enough to excite with adequate energy to lead to the change of redox state of the PQ pool. Therefore ARR10 plays a key role in maintaining homeostasis by regulating chlorophyll biosynthesis to increase the absorption of the portions of light spectrum that are deficient. By increasing the absorption of deficient light, any imbalance in the excitation of energy distribution between PSII and PSI will be counteracted. This further confirms that ARR10 is involved in the long-term photosynthetic acclimation response under different spectral qualities of light.

In Table 1, the connectivity between the TFs AtPHR1 and RAP2.8 is similar to the connectivity between ATHB-1 and ARR10. The connectivity number of AtPHR1 is greater than that of RAP2.8 under the PSI-to-PSII light shift and this ordering is reversed under the PSII-to-PSI light shift. This demonstrates that AtPHR1 and RAP2.8 play different roles under different light shift conditions. Alternatively, one of the transcription factors in Table 1, HY5, does not demonstrate a change of connectivity between the networks of the different light shifts and consequently is not present in two differential networks (Figures 4a and 4b). Since the connectivity is not influenced by the different light shifts, it appears that HY5 may be involved in the inherent process of photosynthesis during the normal light conditions. These results demonstrate that the transcription factors that have been identified play different roles under different light conditions, and that ATHB-1, ARR10, AtPHR1, and RAP2.8 participate in the long term photosynthetic light acclimation response.

A total of 7 regulators are considered as potential *Arabidopsis thaliana *LTR-related TFs, which are predicted from our target gene pool via PlantPAN database. We seek evidence from the literature to validate their inferred function in regulation of the photosynthetic light acclimation response. ATHB-1 is involved in response to blue light [34], while ATHB-2 and HY5 are strongly induced by far-red light [35,36] and involved in red or far-red light signaling pathways [37,38]. This suggests that they are light-related transcription factors and may be related to the photosynthetic light acclimation. Although red and far-red light signaling may be induced by the cytosolic photoreceptor phytochrome, it has not been determined whether signals from photoreceptors and the PQ pool converge to a molecular component under a long term PSI or PSII light conditions. Fey et al (2009) [39] identified photoreceptors that are not essential for adjustment of photosystem stoichiometry, however our results indicate that two signal sources coming from different light systems may share the same TFs to regulate gene expression in the nucleus regardless of whether their signal transductions are coupled or not in the cytosol (Figure 1).

**Figure 6:**
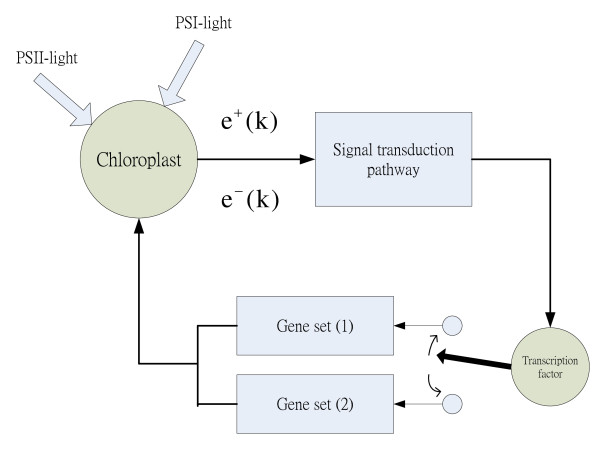
**Feedback control system to mimic the photosynthetic light acclimation mechanism**. Two PS-lights represent high energy input (PSII-light) and low energy input (PSI-light), respectively. *e*^- ^(*k*) denotes the difference between the oxidized state of PQ pool and the homeostatic state. *e*^+ ^(*k*) denotes the difference between the reduced state of PQ pool and the homeostatic state. Chloroplast signals inform the transcription factors how to cope with changes in the surrounding environment via the signal transduction pathway in the cytosol. The transcription factors act as mode switch between different regulating gene sets to display different modes which counteract any change in light input.

According to the results, the transcription factors maintain homeostasis under two different qualities of light by altering regulation of two gene regulatory systems. Figure 6 provides a visualized representation of the photosynthetic light acclimation mechanism as a feedback control system in which the transcription factors function in the role of mode switching. Based on this feedback control system, photosynthetic light acclimation under the two PS-lights can be regarded as a mode switch between two different gene regulatory patterns to improve the efficiency of energy conversion.

We explored The Arabidopsis Information Resource (TAIR) database to obtain annotations of seven TFs in the gene regulatory networks we constructed for this study. Interestingly, five of the seven TFs (ARR10, AtPHR1, ATE2FA, RAP2.8, and ATHB-2) are involved in the leaf senescence stage of *Arabidopsis *[40]. ARR10 and ATHB-1 are involved in the cytokinin-mediated signaling pathway and respond to cytokinin stimulus [33,41]. HY5 is involved in photomorphogenesis [42] and ATHB-1 is involved in the leaf morphogenesis [43]. The diversity of these TFs demonstrates that the long term acclimation response in plants is complicated and is connected to many biological processes. For instance, when a plant is in the shadow of a newly established building, the building can change the redox state of the PQ pool for an extended period of time. This phenomenon will cause the plant to attempt to maintain homeostasis by reconfiguring the relative amount of the two photosystems via this mode switch. If the mode switch is not sufficient to maintain homeostasis, the plant may respond to the change through alternate processes, such as leaf senescence, cytokinin stimulus and altering leaf morphogenesis to construct a new leaf structure. For long-term changes in light quality, the plant will facilitate leaf senescence to remove old leaves and allow new buds to rapidly develop into the leaves with a structure more suitable for the current spectral quality of light. This response is a reconstruction as opposed to a mode switch and is also a light-induced long-term response.

Through analysis of these results, it is possible to predict the important TFs corresponding to opposite redox states of the PQ pool. More biological insight into the gene networks in two different photosystem light shift conditions is given in the discussion section below.

## Discussion

The LTR is initiated whenever a photosynthetic organism is subjected to a stable light gradient for an extended period of time. Because this response can be easily investigated by growing plants in the laboratory under an artificial light source that preferentially excite PSII or PSI (so-called PSII-light or PSI-light systems), the response has been well characterized at the biological level. However, the integration of gene regulation and metabolism during the acclimation process is currently poorly understood. Some studies have attempted to further our understanding of the LTR by identifying primary target genes under different light intensities and spectral qualities [22,27,44-46]. The number of these identified genes that respond to the photosynthetic redox signal in artificial light system can then be tested by observing microarray expression data following mutation of the specific gene or treatment with site-specific electron transport inhibitors. However, the identification of tentative target genes may help only in understanding single aspects of these processes. The full extent to which the LTR controls gene expression in higher plants is still poorly understood and will require systems biology approaches to assess large scale time series microarray data [47].

This study has employed a systems biology approach, combined with a prediction database to construct gene regulatory networks using microarray data. The proposed systematic approach can depict the transcriptional regulatory mechanism of a photosynthesis network as a dynamic system. Furthermore, the time profile of mRNA of each target gene can be described by the dynamic gene regulatory equation in (1). Some researchers have applied similar dynamic equations to construct regulatory networks in yeast and *E. coli *with high accuracy [48,49]. However, they did not consider that the potential regulator set S in equation (1) may include false positive regulators. In this study, we therefore employed maximum likelihood method (see additional file 3) and the Akaike Information Criterion (AIC) [23] via system order detection. To approach the minimum AIC value (see additional file 3), iteratively removing insignificant regulators one by one, the correct regulators of the target gene can be obtained. The parameter estimation method and system order detection method are widely applied in systems biology have been shown to be an efficient tool of reverse-engineering technology.

**Figure 7:**
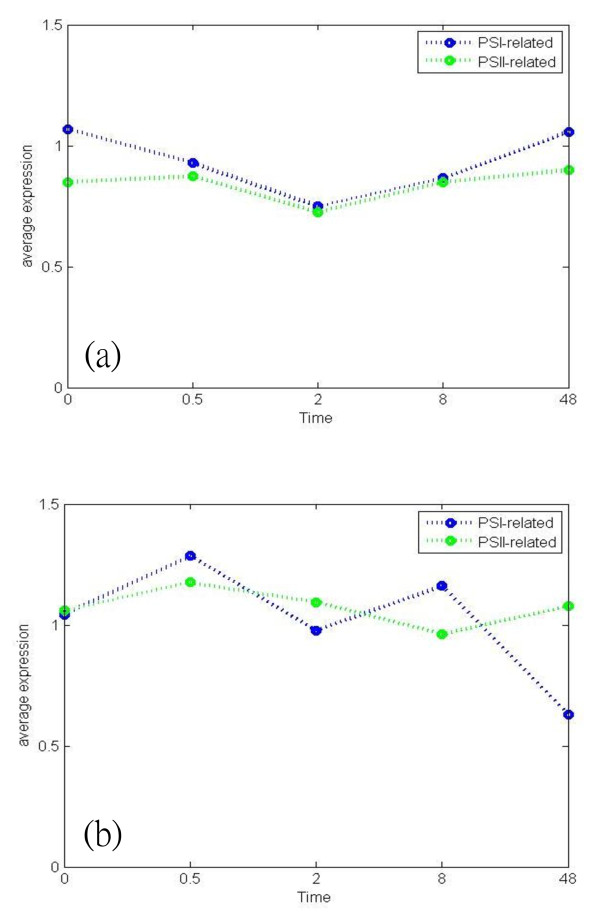
**The average expression of PSII and PSII in two light shift conditions**. (a) represents the average expression of PSI-related and PSII-related genes in reduced state of the PQ pool, (b) represents the average expression of PSI-related and PSII-related genes in oxidized state of the PQ pool. The PSI-related genes consist of genes encoded the photosystem I reaction center subunit and LHCI. The PSII-related genes consist of genes encoded the photosystem II reaction center subunit and LHCII. Every gene must be regulated in differential gene regulatory networks.

The adjustment of photosystem stoichiometry is a long-term response to changed light quality by changing the relative number of the two photosystems [2-6]. Figure 7 shows the average expression of genes encoding each photosystem and its corresponding light-harvesting protein complex present in two differential gene regulatory networks (Figure 4a and 4b), respectively. In Figure 7a, due to the lack of PSI-light, the plant must counteract the reduced state of the PQ pool by increasing the relative expression of PSI-related genes. The average expression of PSI-related genes is greater than that of the PSII-related genes at each time point. In contrast, Figure 7b illustrates that a lack of PSII-light causes the plant to counteract the oxidized state of the PQ pool by increasing the relative expression level of PSII-related genes. In this situation, the average expression of PSI-related genes tends to decrease after 8 h. Moreover, the average expression of PSII-related genes has no significant change in both light shift conditions. This result is consistent with results gathered for mustard (*Sinapis alba *L.) [50] which found that the stoichiometry is regulated by only changing the number of PSI, while PSII remains constant. Although mRNA expression may not exactly represent its corresponding protein concentration, mRNA expression is thought to at least partially reflect protein level [51,52]. Consequently, the results above demonstrate that the constructed networks have a high level of confidence.

Recent studies [1,53] have shown that the STN7 kinase mediates the redox states of the PQ pool. STN7 kinase is regarded as a source of retrograde signals under changes in the spectral quality of light. These light-related retrograde signals are transported across the double membrane of the chloroplast and initiate an unknown signal transduction pathway in the cytosol which carries the signal into the nucleus to affect the expression of nuclear genes (Figure 1). The mechanism by which expression of these genes is altered remains unknown. Furthermore, upstream TFs in the nucleus which are directly activated by a signal from cytosol are also poorly understood. These TFs serve as the interface between the signaling pathway and the gene regulatory network (Figure 1). In step 2 of Stage I, the initial rough regulatory network is developed by iteratively searching for TF-binding sites via the PlantPAN database. Due to the limit of microarray data, the iterative procedure is stopped when no new regulatory interactions are generated from the PlantPAN database. This searching method can identify the TFs which are on the top of overall gene regulatory network and are directly activated by signals from the cytosol. In this study, the gene regulatory networks in Figures 3a and 3b are visualized in a hierarchical form. This shows the stratum of each gene in the network. In Figure 3a, ARR10 is clearly on the top of the gene regulatory network and thus can be considered as the source of the overall gene network for LTR in the nucleus. When the reduced state of the PQ pool is signaled to the nucleus, ARR10 is directly induced. This transduces the signal to the downstream genes to counteract any imbalance in excitation of energy distribution between two photosystems. In Figure 3b, ARR10 is also on the top of gene regulatory network of the oxidized state of the PQ pool. However, it is noteworthy that ARR10 represses ATHB-1 and then ATHB-1 activates ARR10. Therefore, we can hypothesize that both ARR10 and ATHB-1 may be directly induced by the signal of oxidized state of the PQ pool. Because the microarray data is not the genome-wide data, this is only a prediction. If the genome-wide microarray data becomes available, it will be possible to obtain more TFs by our proposed algorithm.

In this study, we use a multi-input/single-output regulatory model to dynamically describe our gene regulatory system. The model analyzes multiple regulators in respect to one target gene, combining time series microarray data to determine the regulatory relationship between this target gene and its upstream regulators. By using Pearson's correlation and the Akaike Information Criterion to prune the complex gene regulatory network, an accurate gene regulatory network of the LTR system in the nucleus can be formed. While our method combines many algorithms to construct a gene regulatory network for the photosynthetic acclimation system, the primary obstacle faced in development of this network is the construction of a rough gene regulatory network. Due to the lack of complete ChIP-chip data for *Arabidopsis thaliana*, the identification of transcriptional regulatory relationships relies on the prediction database. The candidate regulators are selected from the pool of potential regulators typically defined by computational prediction or sequence similarity analysis. If an important regulator is not included in the pool, it will inevitably escape identification by the proposed modeling approach.

## Conclusions

The photosynthetic light acclimation response is a fundamental process in plants which can optimize the efficiency of photosynthesis in fluctuating light quality. However, little is known about the molecular mechanisms that regulate these acclimation responses. In this study, we investigate this molecular mechanism from the physiological level by constructing a gene regulatory network from the limited data sources for *Arabidopsis thaliana *using an efficient computational framework. This type of systems biology approach has become increasingly common in recent years and can provide insight into the underlying mechanisms of the photosynthetic light acclimation response. Gene regulatory networks under PSI-to-PSII light shift and PSII-to-PSI light shift are compared to identify the important transcription factors involved in the regulation of photosynthetic long-term response. The hubs and "weak linkages" are also analyzed for the robust gene network under different light conditions. Although the gene regulatory networks are a small part of photosynthetic light acclimation response in plants, they may provide a foundation on which the overall molecular mechanism underlying the photosynthetic process in organisms may be deciphered. Moreover, it is hoped that the results of this study will attract more attention to the topic of photosynthetic light acclimation with large-scale experiments. As experimental data for whole-genome ChIP-chip data and microarray data with more multiple sampling points become available in the future, the performance of the proposed method will be improved and enable more efficient construction of genome-wide regulatory networks of photosynthetic system. Such genome-wide gene regulatory networks may provide a better understanding for the molecular interaction mechanisms between the chloroplastic, nucleic, and mitochondrial components of the cell under the long term photosynthetic acclimation response. For instance, the regulatory mechanisms for gene network of state transition, a short-term response, in the chloroplast. Furthermore, if more protein-protein interaction data becomes available, the protein interactions of the signal transduction pathway may be identified and integrated with the gene transcriptional regulatory network to provide more insight into the overall photosynthetic light acclimation response.

## Authors' contributions

CWY developed the method, performed the simulation, evaluated the results and drafted the manuscript. BDH provided essential guidance and revised the manuscript. BSC conceived of the study and revised the manuscript. All authors have read and approved the final manuscript.

## Supplementary Material

Additional file 1 **Total 65 target genes**. The Gene annotations for the 65 genes of interest are shown in this table. Click here for file

Additional file 2 **Potential TF-gene pairs of each gene**. The first column represents gene symbols and the second column lists their predictive regulators. Click here for file

Additional file 3 **Supplementary Methods**. Details of system identification methods. Click here for file

Additional file 4 **Connection degree of each node**. The degree of connection for each target gene under PSI-to-PSII and PSII-to-PSI light shifts. Click here for file
