# Supplementary material for: Constructing gene regulatory networks for long term photosynthetic light acclimation in Arabidopsis thaliana
Source: BMC Bioinformatics. 2011 Aug 11;12:335. doi: 10.1186/1471-2105-12-335 (PMC3162938; doi:10.1186/1471-2105-12-335)
Supplement: Additional file 1 — Total 65 target genes. The Gene annotations for the 65 genes of interest are shown in this table. [file 1471-2105-12-335-S1.PDF]

Additional file 1 - Total 65 target genes and their annotation

Gene name	AGI ID	description
ATHB-1	At3g01470	homeobox-leucine zipper protein 5 (HAT5) / HD-ZIP protein 5 / HD-ZIP protein (HB-1)
ARR10	At4g31920	two-component responsive regulator family protein / response regulator family protein
AtPHR1	At4g28610	myb family transcription factor
ATE2FA	At2g36010	E2F transcription factor-3 (E2F3)
RAP2.8	At1g68840	DNA-binding protein RAV2 (RAV2) / AP2 domain-containing protein RAP2.8
ATHB-2	At4g16780	homeobox-leucine zipper protein 4 (HAT4) / HD-ZIP protein 4
HY5	At5g11260	bZIP protein HY5 (HY5)
AB165	At1g29920	chlorophyll A-B binding protein 165/180, chloroplast / LHCII type I CAB-165/180
LHB1B2	At2g34420	chlorophyll A-B binding protein / LHCII type I (LHB1B2)
LHB1B1	At2g34430	chlorophyll A-B binding protein / LHCII type I (LHB1B1)
LHCB2.2	At2g05070	chlorophyll A-B binding protein / LHCII type II (LHCB2.2)
LHCB2.1	At2g05100	chlorophyll A-B binding protein / LHCII type II (LHCB2.1) (LHCB2.3)
LHCB2.3	At3g27690	chlorophyll A-B binding protein (LHCB2:4)
LHCB3	At5g54270	chlorophyll A-B binding protein / LHCII type III (LHCB3)
LHCB4.1	At5g01530	chlorophyll A-B binding protein CP29 (LHCB4)
LHCB4.3	At2g40100	chlorophyll A-B binding protein (LHCB4.3)
LHCB4.2	At3g08940	chlorophyll A-B binding protein (LHCB4.2)
LHCB5	At4g10340	chlorophyll A-B binding protein CP26
LHCB6	At1g15820	chlorophyll A-B binding protein
LHCA1	At3g54890	chlorophyll A-B binding protein / LHCI type I (CAB)
LHCA2	At3g61470	chlorophyll A-B binding protein (LHCA2)
LHCA6	At1g19150	chlorophyll A-B binding protein, putative / LHCI type II
LHCA3	At1g61520	chlorophyll A-B binding protein / LHCI type III (LHCA3.1)
CAB4	At3g47470	chlorophyll A-B binding protein 4
PSAD-2	At1g03130	photosystem I reaction center subunit II
PSAD-1	At4g02770	photosystem I reaction center subunit II
PSAE-2	At2g20260	photosystem I reaction center subunit IV
PSAE-1	At4g28750	photosystem I reaction center subunit IV
PSAF	At1g31330	photosystem I reaction center subunit III family protein
PSAG	At1g55670	photosystem I reaction center subunit V

PSAH-2	At1g52230	photosystem I reaction center subunit VI
PSAH-1	At3g16140	photosystem I reaction center subunit VI
PSAK	At1g30380	photosystem I reaction center subunit psaK
PSAL	At4g12800	photosystem I reaction center subunit XI
PSAN	At5g64040	photosystem I reaction center subunit PSI-N
OEC33	At3g50820	Encodes a protein which is an extrinsic subunit of photosystem II and which has been proposed to play a central role in stabilization of the catalytic manganese cluster
MSP-1	At5g66570	Encodes a protein which is an extrinsic subunit of photosystem II and which has been proposed to play a central role in stabilization of the catalytic manganese cluster
PSBP-2	At2g30790	photosystem II oxygen-evolving complex 23
PPL1	At3g55330	photosystem II reaction center PsbP family protein
PSBP-1	At1g06680	photosystem II oxygen-evolving complex 23 (OEC23)
At3g01440	At3g01440	oxygen evolving enhancer 3 (PsbQ) family protein, photosystem II oxygen-evolving complex protein
PSBQ-2	At4g05180	Oxygen evolving enhancer protein 3 (PsbQ)
PSBR	At1g79040	photosystem II 10 kDa polypeptide PsbR
NPQ4	At1g44575	photosystem II 22kDa protein
PSBW	At2g30570	photosystem II reaction center W (PsbW) protein-related
PSBY	At1g67740	photosystem II core complex proteins psbY
PSB27	At1g03600	photosystem II family protein
At1g80130	At1g80130	expressed protein
At4g19430	At4g19430	expressed protein
At5g11930	At5g11930	glutaredoxin family protein
APL3	At4g39210	glucose-1-phosphate adenylyltransferase large subunit 3 (APL3) / ADP-glucose pyrophosphorylase
PM129	At2g41410	putative calmodulin
PTAC15	At5g54180	mitochondrial transcription termination factor-related / mTERF-related
RFC3	At3g17170	ribosomal protein S6 family protein (RFC3)
At2g40400	At2g40400	expressed protein
At1g69200	At1g69200	pfkB-type carbohydrate kinase family protein
CP29	At3g53460	29 kDa ribonucleoprotein
CS26	At3g03630	O-acetylserine (thiol) lyase
At3g54090	At3g54090	pfkB-type carbohydrate kinase family protein
At2g44040	At2g44040	dihydrodipicolinate reductase family protein
PSAO	At1g08380	expressed protein

PSAP	At2g46820	expressed protein
PSBTN	At3g21055	photosystem II 5 kD protein
PSBQ	At4g21280	oxygen-evolving enhancer protein 3, chloroplast, putative (PSBQ1) (PSBQ)
PSBX	At2g06520	putative membrane protein
