# Supplementary material for: Constructing gene regulatory networks for long term photosynthetic light acclimation in Arabidopsis thaliana
Source: BMC Bioinformatics. 2011 Aug 11;12:335. doi: 10.1186/1471-2105-12-335 (PMC3162938; doi:10.1186/1471-2105-12-335)
Supplement: Additional file 3 — Supplementary Methods. Details of system identification methods. [file 1471-2105-12-335-S3.PDF]

# Supplementary Methods

**Identifying a dynamic model for gene regulatory network via microarray data**

After constructing the stochastic dynamic gene regulatory equation (1) to model the regulation of a target gene by their transcription regulators in the rough gene regulatory network, we use the method of maximum likelihood to estimate the kinetic parameter of dynamic gene regulatory model. Equation (1) can be written in the following form.

$$y[t+1] = \left[ y[t] \;\; x_1[t] \cdots x_L[t] \;\; 1 \right] \bullet \begin{bmatrix} a \\ b_1 \\ \vdots \\ b_L \\ k \end{bmatrix} + \varepsilon[t] \qquad (1)$$

$$= \phi[t] \bullet \theta + \varepsilon[t]$$

where $a = 1 - \beta$, $\phi[t]$ denotes the regression vector that can be obtained from time series microarray data, and $\theta \in R^p$ denotes the parameter vector of dimension $p$ in the regression equation of (1).

After applying the cubic spline method to interpolate the microarray data, we can obtain as many data points as we want. Then it is easy to obtain values of $\{y[t+l] \;\; x_i[t+l] \;\}$ for $l \in \{1, 2, \ldots, m\}$ and $i \in \{1 \;\; 2 \;\; \cdots \;\; L\}$, where m is the number of expression time points of a target gene, and L is the number of TFs binding to the target gene in the rough gene network. By further computation of equation (1) at different time points we can obtain the following vector form equation by data point interpolation.

$$\begin{bmatrix} y[t+2] \\ y[t+3] \\ \vdots \\ y[t+m-1] \\ y[t+m] \end{bmatrix} = \begin{bmatrix} \phi[t+1] \\ \phi[t+2] \\ \vdots \\ \phi[t+m-2] \\ \phi[t+m-1] \end{bmatrix} \cdot \theta + \begin{bmatrix} \varepsilon[t+1] \\ \varepsilon[t+2] \\ \vdots \\ \varepsilon[t+m-2] \\ \varepsilon[t+m-1] \end{bmatrix} \tag{2}$$

For simplicity, it can be represented as follows.

$$Y = \Phi \cdot \theta + e \tag{3}$$

In equation(3), the random noise $\varepsilon[t_k]$ is regarded as a random variable of white Gaussian noise with zero mean and unknown variance $\sigma^2$, i.e., $E\{e\} = 0$, and $\Sigma_e = E\{ee^T\} = \sigma^2 I$, where $I$ is an identity matrix. In this study, a maximum likelihood parameter estimation method is used to estimate $\theta$ and $\sigma^2$ by the regression data obtained from the time series microarray data of regulatory genes and the target gene. Under the assumption of the Gaussian noise vector $e$ with $m-1$ elements, its probability density function is given as follows.

$$p(e) = \frac{1}{((2\pi)^{m-1} \det \Sigma_e)^{1/2}} \exp(-\frac{1}{2} e^T \Sigma_e^{-1} e) \tag{4}$$

From equation (4), we can obtain the likelihood function

$$L(\theta, \sigma^2) = P(\theta, \sigma^2) = \frac{1}{(2\pi\sigma^2)^{(m-1)/2}} \exp\{-\frac{(\mathrm{X} - \Phi \cdot \theta)^T (\mathrm{X} - \Phi \cdot \theta)}{2\sigma^2}\} \tag{5}$$

Equation (5) can be considered as a function of parameters $\theta$ and $\sigma^2$. In order to simplify computation, it is practical to take the logarithm of equation (5), which yields the following log-likelihood function:

$$\log L(\theta, \sigma^2) = -\frac{m-1}{2} \log(2\pi\sigma^2) - \frac{1}{2\sigma^2} \sum_{k=1}^{m-1} [y[t+k+1] - \phi[t+k] \cdot \theta]^2 \tag{6}$$

where $y[t+k]$ and $\phi[t+k]$ are the *k-th* elements of $Y$ and $\Phi$ in (3),

respectively.

By the maximum likelihood parameter estimation method, we expect the log-likelihood function to have its maximum at $\theta = \hat{\theta}$ and $\sigma^2 = \hat{\sigma}^2$. The necessary conditions for the maximum likelihood estimates $\hat{\theta}$ and $\hat{\sigma}^2$ are as follows [24],

$$\begin{aligned} \frac{\partial \log L(\theta, \sigma^2)}{\partial \theta} = 0 \\ \frac{\partial \log L(\theta, \sigma^2)}{\partial \sigma^2} = 0 \end{aligned} \quad (7)$$

The estimated parameters $\hat{\theta}$ and $\hat{\sigma}^2$ are shown below,

$$\hat{\theta} = (\Phi^T \Phi)^{-1} \Phi^T Y \quad (8)$$

$$\hat{\sigma}^2 = \frac{1}{m-1} \sum_{k=1}^{m-1} [y[t_{k+1}] - \phi[t_k] \cdot \hat{\theta}]^2 = \frac{1}{m-1} (Y - \Phi \cdot \hat{\theta})^T (Y - \Phi \cdot \hat{\theta}) \quad (9)$$

where $Y$ and $\Phi$ can be obtained from the time series microarray data of regulatory genes and the target gene in the rough gene regulatory network. After obtaining the estimated parameter $\hat{\theta}$, the dynamic equation of the target gene in the estimated transcriptional regulatory network can be expressed as follows

$$y[t+1] = \hat{a} \cdot y[t] + \sum_{i=1}^{L} \hat{b}_i \cdot x_i[t] + \hat{k} + \varepsilon[t] \quad (10)$$

where $\hat{a}$, $\hat{b}_i$ and $\hat{k}$ are obtained from (8) and the variance is obtained from (9). Iteratively, one target gene at a time, we can construct overall dynamic equations of the transcriptional regulatory network, which are interconnected through the regulations $\sum_{i=1}^{L} \hat{b}_i \cdot x_i[t]$ of TFs.

Since some interaction coefficients $\hat{b}_i$ of the rough gene regulatory network in (10) are insignificant, they should be pruned off by the parsimonious AIC criterion.

This is discussed in the next section.

**Pruning the rough gene regulatory network**

First, in this study, we use the PlantPAN database to identify the plausible TF binding sites roughly and select candidate regulators from the pool of DNA sequence similarity analysis. A rough gene regulatory network is constructed by linking target genes and their possible transcriptional regulators in Step 3. Then we use the maximum likelihood estimation method to estimate the parameters of the dynamic gene regulatory model for a preliminary or rough gene regulatory network of photosynthetic acclimation response.

Although the maximum likelihood estimation method can help us quantify the regulatory abilities of all the possible transcriptional regulation candidates of regulators on target genes of the rough gene regulatory network, we still do not know if the regulatory ability is significant enough that a transcriptional regulator can be regarded as a true regulator of the target gene. In order to determine whether a regulator is significant or not, a statistical approach based on model order validation is proposed for evaluating the significance of our model parameters to prune the preliminary gene network. In this study, a statistical approach called the Akaike Information Criterion (AIC) in [23] is employed to validate the model order (or the number of model parameters) to determine the significance of our dynamic model parameters.

The Akaike Information Criterion (AIC), which attempts to include both the estimated residual variance and the model complexity in one statistic, decreases as the residual variance $\hat{\sigma}^2$ decreases and increases as the number $p$ of parameters increases. As the expected residual variance decreases with increasing $p$ for non-adequate model complexities, there should be a minimum around the correct

number $p$ of network parameters. For a transcriptional regulatory model with $p$ regulatory parameters to fit with data from N samples, the Akaike Information Criterion (AIC) can be written as follows[24],

$$AIC(p) = \log(\frac{1}{N}(Y-\hat{Y})^T(Y-\hat{Y})) + \frac{2p}{N} \quad (11)$$

where $\hat{Y}$ denotes the estimated expression profile of the target gene, i.e. $\hat{Y} = \phi \cdot \hat{\theta}$. This is a tradeoff between residual variance and model order. The minimization of equation (11) will achieve the true model order (i.e. the number of regulators of the target gene) of the gene regulatory system [23].

After the statistical selection of $p$ parameters by minimizing the Akaike Information Criterion (AIC), we can easily determine whether the regulatory TFs candidate is a significant one or just a false positive for a target gene. In the pruning process by AIC, the number of transcriptional regulators of the target gene in **錯誤! 找不到參照來源。** or (10) is reduced from L to $p$. The above parameter estimation and system order detection for dynamic gene regulatory equation in equation **錯誤! 找不到參照來源。** are performed one target gene at a time, and then we can construct a refined gene regulatory network for long-term response of photosynthetic acclimation.
